# The effects of model complexity and size on metabolic flux distribution and control: case study in *Escherichia coli*

**DOI:** 10.1186/s12859-021-04066-y

**Published:** 2021-03-20

**Authors:** Tuure Hameri, Georgios Fengos, Vassily Hatzimanikatis

**Affiliations:** Laboratory of Computational Systems Biotechnology (LCSB), Swiss Federal Institute of Technology (EPFL), 1015 Lausanne, Switzerland

**Keywords:** Metabolic networks, Kinetic model, Metabolic control analysis, Model complexity, Model reduction

## Abstract

**Background:**

Significant efforts have been made in building large-scale kinetic models of cellular metabolism in the past two decades. However, most kinetic models published to date, remain focused around central carbon pathways or are built around ad hoc reduced models without clear justification on their derivation and usage. Systematic algorithms exist for reducing genome-scale metabolic reconstructions to build thermodynamically feasible and consistently reduced stoichiometric models. However, it is important to study how network complexity affects conclusions derived from large-scale kinetic models built around consistently reduced models before we can apply them to study biological systems.

**Results:**

We reduced the iJO1366 *Escherichia Coli* genome-scale metabolic reconstruction systematically to build three stoichiometric models of different size. Since the reduced models are expansions around the core subsystems for which the reduction was performed, the models are nested. We present a method for scaling up the flux profile and the concentration vector reference steady-states from the smallest model to the larger ones, whilst preserving maximum equivalency. Populations of kinetic models, preserving similarity in kinetic parameters, were built around the reference steady-states and their metabolic sensitivity coefficients (MSCs) were computed. The MSCs were sensitive to the model complexity. We proposed a metric for measuring the sensitivity of MSCs to these structural changes.

**Conclusions:**

We proposed for the first time a workflow for scaling up the size of kinetic models while preserving equivalency between the kinetic models. Using this workflow, we demonstrate that model complexity in terms of networks size has significant impact on sensitivity characteristics of kinetic models. Therefore, it is essential to account for the effects of network complexity when constructing kinetic models. The presented metric for measuring MSC sensitivity to structural changes can guide modelers and experimentalists in improving model quality and guide synthetic biology and metabolic engineering. Our proposed workflow enables the testing of the suitability of a kinetic model for answering certain study-specific questions. We argue that the model-based metabolic design targets that are common across models of different size are of higher confidence, while those that are different could be the objective of investigations for model improvement.

**Supplementary Information:**

The online version contains supplementary material available at 10.1186/s12859-021-04066-y.

## Background

Kinetic models of cellular metabolism can provide comprehensive understanding on the dynamics of the cell and its response to environmental changes and perturbations. In depth understanding of cellular metabolism can allow metabolic engineers to tailor cells according to sought specifications and objectives. This could enable the design of cell factories where flux is directed towards the production of biofuels, pharmaceuticals or other specialty chemicals. To be useful though, a kinetic model should represent the dynamics of the cell accurately enough to provide the required study-specific knowledge [1]. To date, important strides towards building large- and genome-scale kinetic models of metabolism have been made [2–5]. Despite the emergence of methodologies for building kinetic models, the research community knows that several challenges remain to be confronted.

With larger and better quality kinetic models, the mathematical representations become increasingly complex. Furthermore, the parameter sensitivities of systems biology models are in general “sloppy” [6]. We have noticed that metabolic models are often built around certain central carbon pathways or, ad hoc reduced models of genome-scale metabolic network models (GEMs) [7]. Such models do not account for the full information contained in the GEMs and, the ad hoc reduced models do not come with explicit explanations and justifications on how the model was reduced. Several studies have built kinetic models around ad hoc reduced models and computed Metabolic Sensitivity Coefficients (MSCs) for the system [1, 2, 8–10]. MSCs are desirable outputs of the kinetic models as they give insight into control patterns of the cell, assuming that the model is correct and accurate. However, Palsson and Lee showed with small-scale models that network complexity significantly affected the numerical values and the interpretation of MSCs [11]. Their study showed that three different red cell metabolic models produced MSCs that have opposite signs. This suggested that the analysis of incomplete metabolic models could lead to misleading and inaccurate information.

It is important to preserve certain level of detail in a metabolic model in order for its predictions to be realistic. The model needs to include important carbon fluxes from the central carbon up to the biomass building blocks, which also includes the synthetic pathway of interest that we would like to engineer. The fact that energy and redox are used by the synthetic pathway of interest makes it important that we account for “all” the reactions and subsystems that carry significant energy and redox fluxes, such as a detailed tricarboxylic acid (TCA) cycle and electron transport chains (ETCs). Keeping such level of detail is essential in order to avoid making false conclusions from kinetic models.

However, nowadays algorithms for reducing GEMs in a more systematic and complete manner are starting to emerge [7, 12–15]. DRUM [14] and MinNW [15] are algorithms that allow the reduction of GEMs but, they do not conserve the feasible flux ranges of the model being reduced. The NetworkReducer algorithm aims to reduce the network around certain “protected” metabolites and reactions by iteratively removing reactions that do not obstruct their activity [13]. Nevertheless, the NetworkReducer does not consider alternative subnetworks that could characterize the GEM being reduced. Ataman et al. developed the redGEM and lumpGEM algorithms which allow reduction of GEMs around selected subsystems by retaining linkages and the information captured in GEMs [7, 12]. The algorithm performs consistency checks with the GEM to ensure that the reduced model is consistent in terms of flux profiles, essential genes and reactions, thermodynamic feasible ranges of metabolite concentrations and ranges of Gibbs free energy of reactions. The redGEM and lumpGEM algorithms can be used to build thermodynamically feasible models with different levels of complexity consistent with the GEM for the same chosen subsystems, whilst considering alternative subnetworks that could be feasible. These algorithms open up the possibility to investigate how MSCs are affected by model complexity for consistently reduced models by building kinetic models around them. For further discussions about available model reduction algorithms, we refer the reader to a recent review [16].

This study investigates the effect of kinetic model complexity – in terms of reaction network size – and its effect on metabolic engineering conclusions derived from MSCs. The elements of complexity that are introduced in the models here are based on the choice of reactions and pathways that are brought into the system before reduction. We used the redGEM and lumpGEM algorithms to reduce the *E. coli* iJO1366 GEM to three different models, namely D1, D2 and D3, encompassing 271, 307 and 327 enzymatic reactions and 160, 188 and 197 metabolites, respectively. The thermodynamic formulation of the stoichiometric models allowed integration of fluxomics and metabolomics data for aerobically grown *E. coli* (see Additional file 1) [17]. Due to the topological differences between the three models, we proposed a technique for scaling up the flux profile and concentration vector reference steady-states from D1 into the larger models D2 and D3. This scale-up procedure ensures physiological equivalency of the models by assuring that their steady-states are numerically similar. All the three models satisfy thermodynamic constraints and are consistent with the GEM. We used the Optimization and Risk Analysis of Complex Living Entities (ORACLE) workflow to construct populations of kinetic models for D1, D2 and D3 around their scaled reference steady-states. Due to the uncertainty in the kinetic parameters, their largely unknown ranges and the high-dimensionality nature of the system, there are multiple possible parameterizations of the kinetic models. Hence, we consider populations of kinetic models in order to account for the multiple possible scenarios arising from uncertainty in parameterization, hereby reducing bias. We fixed kinetic parameters from the smaller model into the larger one to further ensure equivalency of the models and hence a fair comparison. As integral part of the ORACLE workflow, we compute the MSCs for the stable kinetic models. The nested nature [18, 19] of the reduced models allows us to methodically compare the MSCs across the three models. We demonstrate that, even when the model preserve certain minimum assumptions of the real world biological system, MSCs are sensitive to model complexity.

The methodology presented in this manuscript allowed us to study the sensitivity of systematically reduced models of aerobically grown *E. coli*. The models were specifically designed to be equivalent variants representing the central carbon metabolism, with only incremental changes in the model size that come along as we extend the level connectivity of the involved subsystems. However, this approach could be applied to models reduced in an ad hoc fashion, as well as other physiologies and organisms. The metrics presented can be used to assess the adequacy of a network for metabolic engineering based on MSCs. If the conclusions derived from MSCs—relevant for strain design of a given biological system—are very sensitive to model complexity, the modeler can decide to improve/reassess the model. Hence, this pipeline can serve as a tool to test and ameliorate model quality for metabolic engineering applications.

## Results

### Reduced *E. coli* models

**Fig. 1:**
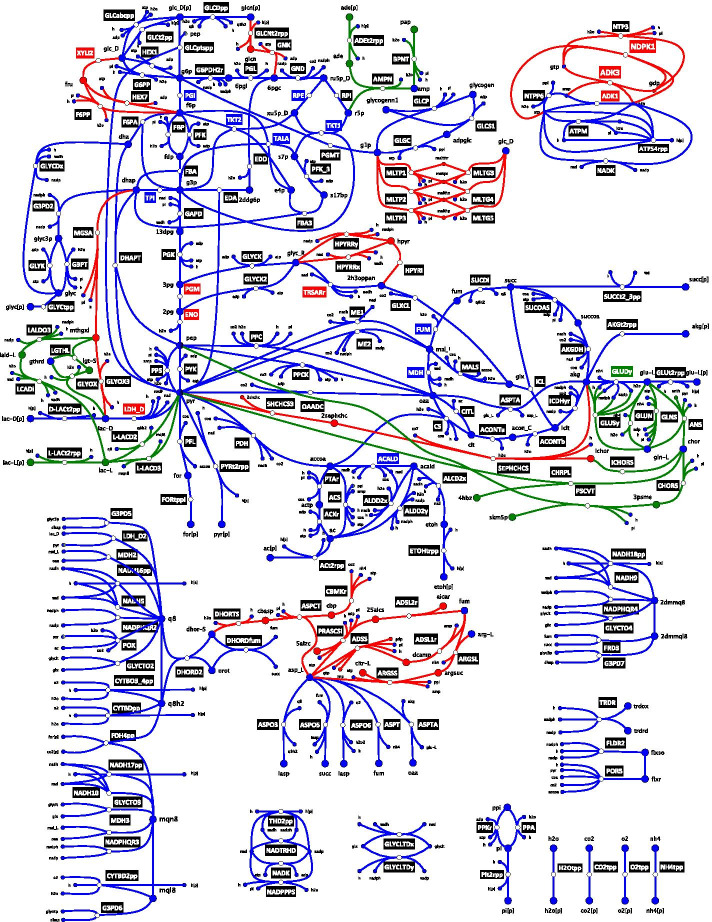
D1, D2 and D3 *E. coli* network diagram illustrating differences in their topologies. D1, D2 and D3 models are constituted of 271, 307 and 327 enzymatic reactions and 160, 188 and 197 metabolites, respectively. The reactions (edges) and metabolites (nodes) are coloured according to their pertinence to D1 (blue), D2 (red) and D3 (green). Reaction labels indicate if a reaction is unidirectional (black) or, bidirectional in D1 (blue), D2 (red) and D3 (green). The reactions that are bidirectional in a smaller model were also bidirectional in the larger models. Diagram does not include all the reactions of the systems. Full details of the reactions and metabolites in the models are provided in Additional file 3. yEd Version 3.20.1 was used to generate the network diagram

**Fig. 2:**
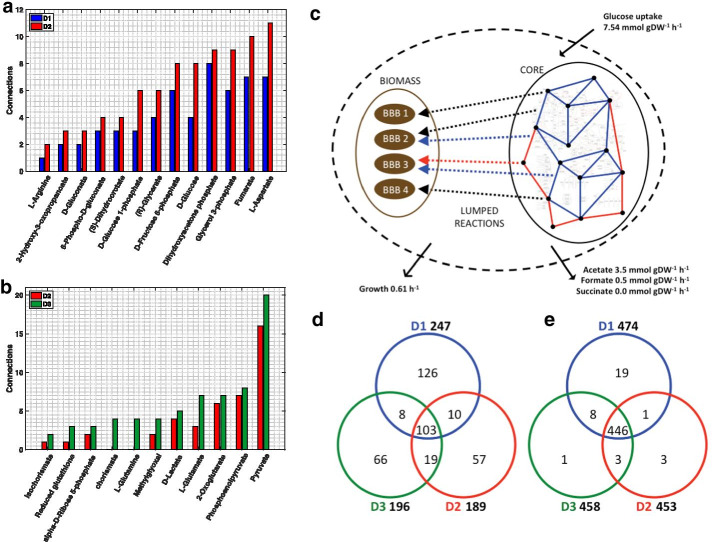
Illustration and analysis of D1, D2 and D3 network topologies. The redGEM algorithm was used to generate D1 (blue), D2 (red) and D3 (green) core enzymatic reaction networks composed of 271, 307 and 327 reactions, respectively. Core network metabolites that change in connectivity (**a**) between D1 and D2, and (**b**) between D2 and D3 are highlighted. These additional connections/reactions result in increased flexibility of the network. The schematic representation (**c**) of the studied metabolic networks shows the reactions (edges) and metabolites (nodes), and how they are connected via lumped reactions (dashed line) to biomass building blocks (brown ellipsoid). There are 102 biomass building blocks (listed in Additional file 3) in the *E. coli* iJO1366 that are preserved across reduced models. Reactions from D1 (blue) and D2 (red) correspond to the core of the metabolic models. The lumped reactions can be unique to D1 (blue) or D2 (red), or be common between both (black). Fluxomics data (black solid arrows) were integrated for optimally grown *E. coli* [17]. Each lumped reaction is composed of multiple reactions lumped together, also referred to as a subnetwork. Venn diagrams highlight differences in the lumped reactions of D1, D2 and D3 in terms of (**d**) subnetworks and in terms of (**e**) reactions composing them

We applied redGEM and lumpGEM algorithms [7, 12] to systematically derive nested, reduced, *E. coli* stoichiometric models (Methods) from the iJO1366 GEM [20]. We selected glycolysis, pentose phosphate pathway (PPP), tricarboxylic acid (TCA) cycle, glyoxylate cycle, pyruvate metabolism and electron transport chain (ETC) as the subsystems (as defined in the iJO1366 GEM [20]) around which reduction was performed to different degrees of connection D, similarly to Ataman et al*.* [7]. D corresponds to the distance between pairs of selected subsystems. The selected subsystems contain the 12 essential biomass precursors defined by Neidhart et al. [21] and capture the central carbon metabolism of *E. coli*. Reduced stoichiometric models D1, D2 and D3 inter-connect the pairs of subsystems with up to one, two and three reactions, respectively (Fig. 1). Hence, the reactions added by the expansions are entirely based on graph-search. The D1, D2 and D3 cores were connected to biomass production via lumped reactions, generated by the lumpGEM, to characterize the rest of the GEM (further discussion on lumped reactions around Fig. 2 later in this section).

The additional reactions in D2 include xylose isomerase (XYLI2), hexokinase d-fructose (HEX7) and d-fructose 6-phosphate phosphatase (F6PP), that connect d-glucose with d-fructose 6-phosphate via d-fructose. D2 also includes the maltodextrin system which connects the d-glucose to d-glucose 1-phophate via the maltodextrin phosphorylase and maltodextrin glucosidase reactions. In D2, dihydroxyacetone phosphate can react to methyglyoxal, which in turn can react to d-Lactate, providing increased connectivity between glycolysis and the pyruvate node. Additionally, pyruvate can react to 2-succinyl-5-enolpyruvyl-6-hydroxy-3-cyclohexene-1-carboxylate, which can react to form 2-oxoglutarate, thus connecting the TCA cycle with the pyruvate metabolism. D2 also includes three different ways to connect with two reactions from fumarate to l-aspartate, which—via argininosuccinate, adenylsuccinate and adenylosuccinate—further link the TCA cycle with the ETC. The adenylate kinase (ADK3), nucleoside-diphosphate kinase (NDPK1) and nucleoside-triphosphatase (NTP3) enzymes provide D2 model with additional flexibility in the system’s energy metabolism.

D3 has additional reactions enabling the transformation of methylglyoxal into d-lactate and l-lactate. Methylglyoxal is a hub metabolite that provides connectivity between upper glycolysis to the pyruvate node. The pyruvate and phosphoenolpyruvate nodes are connected to the TCA cycle via chorismate. Fruthermore, the glutamine and glutamate synthases provide additional flexibility in allowing conversion between l-glutamate and l-glutamine. In D3 the presence of AMP nucleosidase (AMPN) provides an additional connection between the PPP and the ETC. However, the expansion from D1 to D2 resulted in more central carbon metabolites that change in connectivity (Fig. 2A) than the expansion from D2 to D3 (Fig. 2B). Several hub metabolites like methylglyoxal, isochorismate, pyruvate, d-lactate and 2-oxoglutarate change in connectivity between the three models.

### Thermodynamic-based variability analysis

Within the thermodynamic formulation [22] of the stoichiometric models D1, D2 and D3, we integrated fluxomics and metabolomics data for aerobically grown *E. coli* (see Additional file 2). Several assumptions were made on reaction directionalities, based on literature [17, 23–26], to further constrain the models (Methods). We performed a thermodynamic-based variability analysis (TVA) [27] on D1, D2 and D3 and we found they had 9, 17 and 18 bi-directional reactions, respectively (see Additional file 3). We noticed that allowable TVA ranges for fluxes and concentrations appeared to differ more between D1 and D2, than between D2 and D3. These differences generally occurred around regions where the network expansion added new branching points. For further discussions on this topic, we refer the reader to our supporting documentation (see Additional file 4).

### Model equivalency

Despite the inclusion of omics data for aerobically grown *E. coli*, D1, D2 and D3 remained underdetermined systems, resulting in the existence of multiple alternative steady-states that can characterize the studied *E. coli* physiology. A representative steady-state is required for the flux profile and for the metabolite concentration vector, to build a kinetic model around the selected steady-state. Furthermore, in light of benchmarking the outputs of kinetic models, the models are required to themselves be as equivalent to each other as possible to allow for an unbiased comparison. Hence, their representative steady-states were kept similar so that the models describe the same operational state of the cell.

#### Scaling up steady-states

We sampled the flux and the concentration solution spaces for D1 and we used PCA to select representative steady-states (Methods). To preserve equivalency across the kinetic models, it was desirable that the flux profile and the concentration vector steady-states in D2 and D3 resemble the ones selected in D1. The nested nature of the core models generated with redGEM eased the transferability of steady-states across models, allowing us to preserve similar values for fluxes and concentrations for the overlapping reactions of the three models.

We connected the core models to the biomass building blocks (BBBs), as defined by Neidhart et al*.* [21], via lumped reactions generated with the lumpGEM algorithm by applying approaches developed by Ataman and Hatzimanikatis [12]. A lumped reaction is a reaction that collapses a subnetwork of reactions into one mass-balanced reaction. D1–3 had 247, 189 and 196 lumped reactions, respectively. The models’ lumped reactions are indeed not the same across D1–3. Consequently, lumped reactions impose certain stoichiometric constraints that can require flux to pass through alternative metabolic routes within the models. For instance, a BBB can be produced by a completely different lumped reaction (Fig. 2C), as we can generate it via a different subnetwork of reactions in the systems with larger cores. Thus, having distinct lumped reactions results in the redistribution of the flux profiles across models. An example of this is the hub metabolite methylglyoxal that provides new alternatives for lumped reactions in D2 and D3, thus contributing to differences in flux distribution across the models.

We studied the lumped reactions in D1–3 and observed that 103 were common between the models (Fig. 2D). D1, D2 and D3 have 126, 57 and 66 lumped reactions that are unique to themselves. D1 requires considerably more lumped reactions in order to produce the BBBs from the core subsystems. If we consider the lumped reactions as subnetworks of reactions, 474, 453 and 458 reactions are used to build the lumped reactions of D1–3, respectively (Fig. 2E). Interestingly, 446 reactions are common between the pools of reactions that constitute the lumped reactions of D1–3. It may appear unexpected that D3 had more lumped reactions than D2. However, this can occur when more “shorter” lumped reactions—that are composed of lesser reactions—are required to produce a given BBB.

In order to ensure equivalency between D1–3, we proposed a procedure that uses a Mixed Integer Linear Programming (MILP) formulation that imposes similarity between the representative steady-states of the models (Methods). The D2 fluxes of central carbon reactions are within below one percent deviation from the reference flux of D1 (see Additional file 5), except for the exporter of d-Alanine (DALAtex) that deviates by 8% (Table 1). The only central carbon fluxes in D3 that deviate from D2 reference flux with more than one percentage are transaldolase (TALA) and xylose isomerase (XYLI2) with 4.5% and 33.2% respectively (Table 1). Other larger deviations occur in transport (periplasm to cytoplasm and extracellular to periplasm) reactions that carry a considerably lower flux such as transporters of l-serine, succinate, l-tryptophan, l-tyrosine, l-valine and zinc (Table 1).

**Table 1:** Deviations in metabolic fluxes between pairs of models

Comparison: model A/model B	Reaction	Absolute % deviation	Flux [mmol/gDW/h]	
			Model A	Model B
D1/D2	DALAtex	8.2	− 1.32E−02	− 1.43E−02
D2/D3	SEPHCHCS	295.6	7.76E−04	3.07E−03
D2/D3	SERt2rpp	2747.2	− 3.59E−04	− 1.02E−02
D2/D3	SERtex	2747.2	− 3.59E−04	− 1.02E−02
D2/D3	SHCHCS3	295.6	7.76E−04	3.07E−03
D2/D3	SO4t2pp	22.6	2.71E−01	3.32E−01
D2/D3	SO4tex	1.0	3.56E−01	3.53E−01
D2/D3	SPMDt3pp	1.2	3.66E−04	3.62E−04
D2/D3	SPMDtex	1.2	− 3.66E−04	− 3.62E−04
D2/D3	SUCCt2_2pp	75.3	4.01E−01	9.90E−02
D2/D3	SUCCt2_3pp	396.8	7.64E−02	3.80E−01
D2/D3	SUCCtex	464.1	− 5.08E−05	− 2.87E−04
D2/D3	SULabcpp	76.4	8.48E−02	2.00E−02
D2/D3	TALA	4.5	2.08E−01	2.17E−01
D2/D3	THD2pp	3.6	6.41E−01	6.64E−01
D2/D3	TRPt2rpp	77.6	− 2.40E−03	− 5.39E−04
D2/D3	TRPtex	77.6	− 2.40E−03	− 5.39E−04
D2/D3	TYRt2rpp	620.7	− 5.69E−04	− 4.10E−03
D2/D3	TYRtex	620.7	− 5.69E−04	− 4.10E−03
D2/D3	VALt2rpp	331.3	− 6.20E−04	− 2.67E−03
D2/D3	VALtex	331.3	− 6.20E−04	− 2.67E−03
D2/D3	XYLI2	33.2	9.19E−03	1.22E−02
D2/D3	ZN2tpp	4.8	1.89E−04	1.80E−04
D2/D3	ZNabcpp	478.5	1.91E−06	1.11E−05

The concentration profile of D2 is within one percent of D1 reference steady-state, except for ADP, CoA, S-dihydroorotate and l-glutamine with 16%, 45%, 303% and 94% deviations from D1 (Table 2). On the other hand, the D3 metabolite concentration steady-state is within one percentage from the D2 metabolite concentration vector. The nested nature and the consistency of redGEM and lumpGEM algorithms in GEM reduction allowed the steady-states to be transferred and communicated between models efficiently.

**Table 2:** Deviations in metabolite concentrations between pairs of models

Comparison: model A/model B	Metabolite	Absolute % deviation	Concentration [log(mM)]	
			Model A	Model B
D1/D2	ADP	16.1	− 10.00	− 8.39
D1/D2	Coenzyme A	45.2	− 8.21	− 11.93
D1/D2	(S)-Dihydroorotate	303.2	− 3.41	− 13.75
D1/D2	l-Glutamine	94.1	− 6.72	− 13.04

#### Equivalence in kinetic parameters

We constructed kinetic models around the selected reference steady-states of D1–3 using the ORACLE workflow [3, 28–30]. Uniform Monte Carlo sampling of the degrees of saturation of the enzyme active sites allowed us to study the kinetic parameter space, as proposed by Wang et al. [28]. The local stability of the models generated was tested by verifying that the eigenvalues are not positive. We first sampled 50,000 stable kinetic models for D1. To ensure equivalency at kinetic parameter level between D1–3, we adapted the ORACLE workflow to allow fixing the sampled saturation states from one model to another (Methods). From the 50,000 stable D1 kinetic models, we found 96.1% (48,080) to be stable in D2, of which 98.4% (47,299) were stable in D3. We then computed the MSCs for these stable models in order to compare how MCA-based decisions are affected by metabolic network size.

### Consistency in MCA across models

#### Ranking enzymes for flux control

**Fig. 3:**
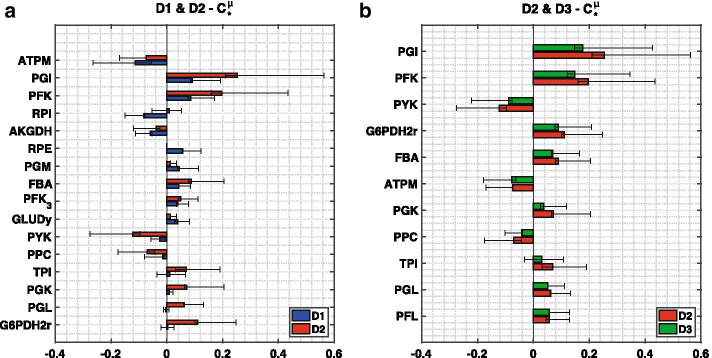
Top enzymes controlling cellular growth (*μ*) across models. The top 9 enzymes based on absolute mean control over cellular growth were computed for D1 (blue), D2 (red) and D3 (green). We then selected the pairwise union of these enzymes for the comparisons of **a** D1 versus D2, and **b** D2 versus D3. The whiskers give the upper and lower quartiles of the FCC populations and the bars give the means

Some fundamental cellular tasks for a given physiology include metabolite excretion, substrate uptake and cellular growth, *μ*. As we studied the physiology of optimally grown *E. coli*, we considered control over *μ* across models to assess the consistency in conclusions based on MSCs. The flux control coefficients (FCCs) of *μ* were ranked for D1–3 based on their absolute means across stable models. The models were compared pairwise in increasing order of size (i.e. D1 versus D2, and D2 versus D3) to assess the impact of systematic network expansion on MSCs (Fig. 3).

The cellular growth FCCs with respect to glucose-6-phosphate isomerase (PGI), phosphofructokinase (PFK) and ATP maintenance (ATPM) are the most consistent in terms of sign and magnitude when comparing D1 with D2 (Fig. 3A). Pyruvate kinase (PYK), fructose biphosphate aldolase (FBA) and 2-oxogluterate dehydrogenase (AKGDH) are also in agreement in terms of sign but magnitude can differ significantly. Some enzymes have control in D1 but no control in D2, such as ribulose 5-phosphate 3-epimerase (RPE) and phosphoglycerate mutase (PGM). Others, vice versa, have control in D2 but no control in D1 such as phosphoglycerate kinase (PGK) and glucose 6-phosphate dehydrogenase (G6PDH2r). Ribose-5-phosphate isomerase (RPI), on the other hand, has opposing control on cellular growth in the two models. Differences in FCCs of cellular growth between D1 and D2 suggest that the expansion of D1 to D2 significantly affects the control scheme. When we compare FCC values pairwise between D1 and D2, we note that numerical values can be relatively dissimilar (see Additional file 4 for supporting information). Hence, differences can still be observed after preserving model equivalency.

We then compared top cellular growth FCCs in D2 and D3, which are in great sign and magnitude agreement (Fig. 3B). PGI, PFK and PYK are the top three enzymes in terms of cellular growth control according to both D2 and D3. The consistency between these FCCs suggests that the expansion of D2 to D3 does not affect the control pattern as significantly as the network expansion from D1 to D2. An analogous analysis was carried out for the flux control of glucose uptake and, the excretions of acetate and formate (see Additional file 4 for supporting information), and we observed a similar trend. The differences in control patterns appear to be more significant when expanding from D1 to D2, but of lesser importance when expanding from D2 to D3. This finding could suggest that entire genome-scale kinetic models are not necessary to capture the essential physiological features of a cell as long as the model reduction is done systematically around carefully selected subsystems that are pertinent to the study. However, this could also mean that D1 is possibly missing on some information for performing MCA around growth. It is difficult to draw more conclusions as we can only compare what is topologically shared between two models. Clearly, a study-specific resolution criterion in terms of model size/complexity that has to be met needs to be established before a model is used for further analysis.

#### MCA consistency across reduced models

As the study above revealed, certain flux control patterns can change significantly between models due to network complexity. We tried to locate, analyze and understand the differences and the similarities in MSCs that occur due to the topological alterations in kinetic model complexity. According to MCA theory, the FCCs conform with the summation theory [31, 32]. We proposed a deviation index (DI) that provides a quantitative measure on how much a reaction’s FCCs differ between two models, postulated from the summation theory (Methods). The DI served as a metric to classify reactions with respect to their consistency in FCCs across the reduced models.

**Fig. 4:**
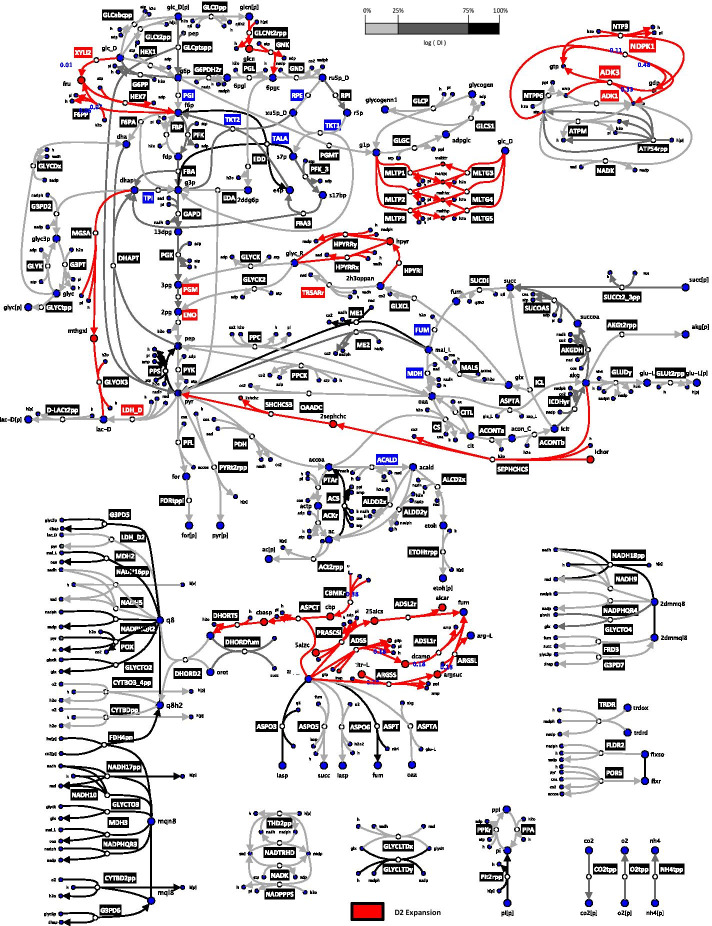
*E. coli* network diagram illustrating the logarithm of the deviation index (DI) of enzymatic reactions when scaling up from D1 to D2. Network of core reactions (edges) and metabolites (nodes) for D1 and D2 models. The DI is an indicator of difference in the control over a reaction with respect to all the enzymatic reactions of the network due to the network expansion (Methods). Reactions added by the redGEM expansion from D1 to D2 (red), and ones in common between D1 and D2 for low (0–25 percentile) DI (light gray), medium (25–75 percentile) DI (dark gray) and high (75–100 percentile) DI (black) are shown. The blue metabolites are common between D1 and D2, and red ones indicate metabolites resulting from the D2 expansion. Diagram does not include all the reactions of the systems. yEd Version 3.20.1 was used to generate the network diagram

We estimated the DI of 271 common enzymatic reactions when expanding from D1 to D2 to predict deviations in FCCs for the system. Reactions with the lowest DI (0–25 percentile) were mostly from the central carbon metabolism (Fig. 4). The reactions with the highest DI (75–100 percentile) were mostly located in the ETC. The only central carbon metabolism reactions having a high DI were TALA, acetyl-CoA synthase (ACS), phosphoenolpyruvate synthase (PPS) and NAD malic enzyme (ME1). TALA produces d-fructose 6-phosphate and, PPS and ME1 involve transformation of pyruvate. d-Fructose 6-phosphate and pyruvate are both central carbon metabolites around which the expansion adds reactions (Figs. 2, 4). ACS is only one reaction away topologically from pyruvate, around which the expansion adds a reaction (Figs. 2 and 4).

**Fig. 5:**
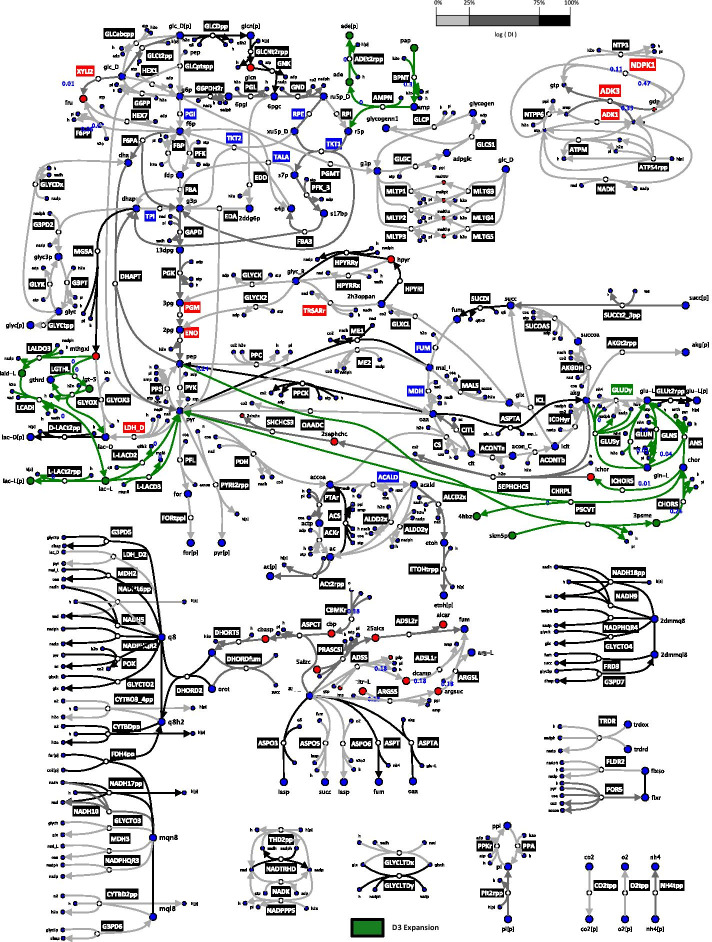
*E. coli* network diagram illustrating the logarithm of the deviation index (DI) of enzymatic reactions when scaling up from D2 to D3. Network of core reactions (edges) and metabolites (nodes) for D2 and D3 models. The DI is an indicator of difference in the control over a reaction with respect to all the enzymatic reactions of the network due to the network expansion (Methods). Reactions added by the redGEM expansion from D2 to D3 (green), and ones in common between D2 and D3 for low (0–25 percentile) DI (light gray), medium (25–75 percentile) DI (dark gray) and high (75–100 percentile) DI (black) are shown. The blue, red and green nodes correspond to metabolites common between D1 and D2, ones added by the D2 expansion and ones added by the D3 expansion, respectively. Diagram does not include all the reactions of the systems. yEd Version 3.20.1 was used to generate the network diagram

We repeated the above analysis for D2 and D3, where we analogously compute the DIs for the 307 common enzymatic reactions (Methods). Similar observations were made for the reactions having low DIs (0–25 percentile) as most were located in central carbon metabolism, within the subsystems around which reduction was performed (Fig. 5). The reactions with higher DIs (75–100 percentile) are predominantly located around the ETC, with the exception of several reactions pertaining to central carbon metabolism. As with the previous analysis of D1 versus D2, ME1 and ACS had high DIs. The D3 expansion adds reactions around pyruvate (Figs. 2, 5), which could explain this observation. Aspartate transaminase (ASPTA), phosphoenolpyruvate carboxykinase (PPCK) and succinate dehydrogenase (SUCDi) from the central carbon metabolism exhibited high DIs (Fig. 5). ASPTA is directly connected via 2-oxoglutarate with NADPH glutamate synthase (GLUSy), which is a newly added reaction by the D3 expansion. PPCK is connected via polyenolpyruvate to 3-phosphoshikimate 1-carboxyvinyltransferase (PSCVT), another add by the D3 expansion. Furthermore, SUCDi is topologically connected with the added reaction ubiquinone l-Lactate dehydrogenase (l-LACD2), as cofactors ubiquinone-8 and ubiquinol-8 partake in both reactions. Interestingly, periplasmic glucose dehydrogen (GLCDpp), where ubiquinone-8 and ubiquinol-8 also participate, has a high DI as well. GLCDpp possibly causes its neighbouring reactions gluconokinase (GNK) and d-gluconate transport (GLCNt2rpp) to have high DIs too, due to stoichiometric coupling. These observations suggest that alterations in flux split ratios around important branching points—caused by network expansion—could result into higher DIs in reactions at their vicinities.

#### Importance of flux splitting nodes

Overall, lower DIs were observed for reactions having a higher flux, pertaining to the core central carbon metabolism around which the models D1–3 were reduced (Figs. 4, 5). Since the cores of the reduced models contain the 12 precursor metabolites for biomass, their control patterns were expected to be similar. Stephanopoulos and Vallino point out that metabolic pathways of organisms have evolved over time to resist flux alterations at branching points [33]. The control architecture of an organism is built such that it preserves the flux splitting ratios of essential metabolic nodes. However, if two models have differences in the number of reactions and/or in the flux splitting ratios around an important branching point, the control architecture of the two systems can differ considerably.

Since we studied optimally grown *E. coli*, it was expected that the D1 to D2 expansion with the addition of XYLI2, F6PP and HEX7 would have influence on control patterns: the flux splitting ratios around the essential biomass precursor d-fructose 6-phosphate is altered. d-Fructose 6-phosphate is a critical metabolic node for producing cell wall biomass building blocks and is located relatively upstream in the process of glucose catabolism. Altering flux splitting ratios around d-fructose 6-phosphate will have direct implications on the fate of the carbon flow across the whole network, particularly due to its upstream location.

The expansion from D2 to D3 results in different flux splitting ratios around three biomass precursors: pyruvate, polyenolpyruvate and 2-oxoglutarate. Again, we can expect flux control patterns across the models to differ as the proportion of carbon flow directed towards certain biomass building blocks is affected. However, within the central carbon metabolism, these precursors are located relatively downstream to the glucose uptake, compared to for instance d-fructose 6-phosphate. Consequently, we can expect that these flux splitting ratios have less impact on the growth control of the system than d-fructose 6-phosphate. If we were discussing the production of certain amino acids of interest, rather than just cellular growth, these ratios could be of higher importance to the analysis. The significance of a metabolic node is strongly subject to the scope of the study. Hence, it is difficult to imagine a “one-size-fits-all” model due to the complexity of the problems encountered in metabolic engineering.

Indeed, the importance of a metabolic branching point is very study-specific as objectives can vary significantly. Had we, for instance, been interested in the study of d-lactate production, it would have been essential to include the metabolism of methylglyoxal, d-lactate and l-lactate into the subsystems around which model reduction is performed. However, as we are not interested in the production of d-lactate, we are not that concerned about the high DI of d-lactate transporter (d-LACt2pp) when comparing D2 and D3 (Fig. 5). Furthermore, if we were interested to produce d-lactate, it would be essential to consider implication of attempting to deviate flux towards the metabolism of d-lactate. If the redirection of flux towards d-lactate imposes important changes in the flux splitting ratios of significant metabolic nodes of wild-type *E. coli*, it may be worth considering other organisms that cause fewer modifications in flux distribution [33, 34].

#### Study of uncertainty in MCA

**Fig. 6:**
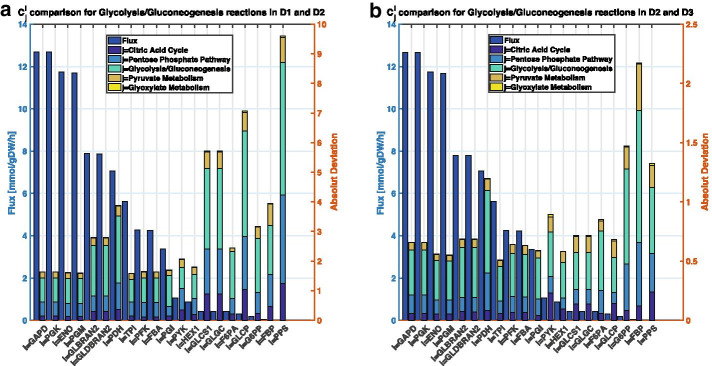
Comparison of flux and absolute deviations in FCCs for glycolytic reactions for **a** D1 versus D2, and **b** D2 versus D3. The absolute deviations computed subsystem-wise (stacked bar) correspond to the sum of the absolute deviations in FCCs of reaction *i* with respect to all enzymes of the subsystem *j.* The reactions contained in a subsystem are as defined in the original GEM that was reduced [20]. The flux values (blue bar) did not deviate by more than 1% between pairs of models

The MSCs of D1–3 were further studied by comparing their absolute deviations in the FCCs. We considered with respect to the central carbon subsystems to find which central carbon enzymes contributed in most uncertainty across the networks. The FCCs of reactions in the glycolysis (Fig. 6A, B) appear to have most absolute deviation stemming from enzymes in the glycolysis and in the PPP. In both comparisons (Fig. 6A, B), glycolysis contributes the most to this deviation. However, in the expansion from D2 to D3 (Fig. 6B), this contribution to the deviation is of a considerably smaller magnitude than in the expansion from D1 to D2 (Fig. 6A). Again, the additional connections around d-fructose 6-phosphate (Figs. 1 and 2) when expanding from D1 to D2 could explain this. Differences in flux splitting ratio around d-fructose 6-phosphate affect the redistribution of the flux in the network and hence the control pattern. Generally, reactions with a larger flux exhibit less absolute deviations in their FCCs. This parallels the observation that central carbon reactions carrying higher flux are perhaps more rigid in control patterns.

**Fig. 7:**
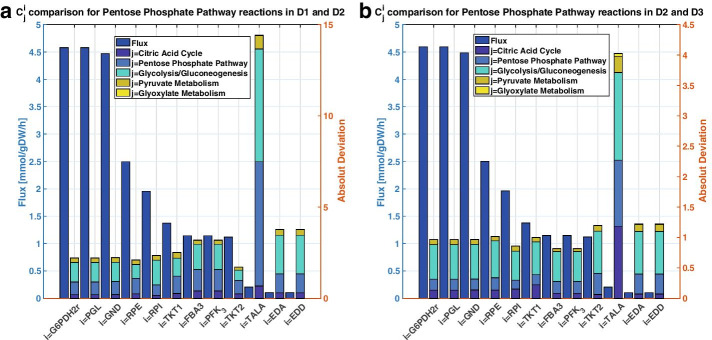
Comparison of flux and absolute deviations in FCCs for PPP reactions for (**a**) D1 versus D2, and (**b**) D2 versus D3. The absolute deviations computed subsystem-wise (stacked bar) correspond to the sum of the absolute deviations in FCCs of reaction *i* with respect to all enzymes of the subsystem *j.* The reactions contained in a subsystem are as defined in the original GEM that was reduced [20]. The flux values (blue bar) did not deviate by more than 1% between pairs of models

We perform a parallel analysis on FCCs of PPP reactions and similar observations were made. In the expansions from D1 to D2 (Fig. 7A) and D2 to D3 (Fig. 7B), glycolysis contributed the most to the absolute deviation of the FCCs of PPP reactions. However, the contribution of the glycolysis enzymes was considerably smaller in magnitude in the expansion from D2 to D3 (Fig. 7B) than in the one from D1 to D2 (Fig. 7B). Again, reactions carrying higher flux have less absolute deviation in their FCCs between the pairs of models. We analyzed FCCs individually in terms of absolute deviation (see Additional file 6), for both pairs D1 and D2 as well as, D2 and D3. PGI, TPI and PFK were the top three central carbon enzymes that resulted in the most absolute difference in flux control across the network. From the PPP enzymes, RPI resulted in the most absolute deviation in flux control. We also recall that RPI had sign-wise opposing control on cellular growth in the comparison of D1 and D2 (Fig. 3A). Due to the highly non-linear nature of the studied systems, it is difficult to make direct conclusions on the causality of the observed deviations in control patterns of the networks. Most of the deviations were observed amongst peripheral transport reactions, rather than central carbon metabolism (see Additional file 6). Nevertheless, we could still find metabolic engineering decisions relevant to our study, independent of the complexity based on MCA outputs (Fig. 3).

## Discussion

Using a kinetic model that is lacking adequate complexity level can result in modelers making false prediction. However, systematic assessment of the impact of model complexity on the conclusions derived from a kinetic model of metabolism has not been carried out in literature to date. Many degrees of freedom exist as the exact metabolic flux distribution, metabolite concentration levels, kinetic mechanisms and the required kinetic mechanism parameters are not fully characterized for biological systems. This multiplicity in sources of uncertainty makes it difficult to study the impact of model complexity on kinetic model conclusion. Hence, model equivalence has to be preserved in order to study the effects of network size on these conclusions. Additionally, certain minimum level of model complexity, such as network parts carrying important carbon flux and redox potential, is required in order for the model predictions to be realistic. We hereby address these issues by demonstrating the effect of network size on conclusions derived from systematically reduced kinetic models, whilst conserving maximum model equivalence.

In this work we study the impact of model complexity on the metabolic engineering decisions derived from MSCs. The redGEM and the lumpGEM algorithms were used to consistently reduce the *E. coli* iJO1366 GEM. Omics data for the physiology of optimally grown *E. coli* was integrated into the reduced stoichiometric models. The nested nature of the reduced models assisted us in the development of a workflow allowing to preserve maximum equivalence between the flux profile and metabolite concentration steady-states. The ORACLE framework was used to generate populations of stable kinetic models around these reduced stoichiometric models. Our workflow ensured that we preserve equivalency amongst the populations of the kinetic parameters for the stable kinetic models. The MSCs were computed within the ORACLE framework for the populations of stable kinetic models. Analysis of the MSCs, revealed that we can derive context-specific metabolic engineering conclusions that are independent of the model’s complexity, as long as the reduction is performed consistently.

The “usefulness” of a kinetic model is highly dependent on the objectives of the study being undertaken. We selected the subsystems for the GEM reduction such that we: (1) cover the essential biomass precursor metabolites according to Neidhart as we focused primarily on cellular growth control and, (2) that we capture the ETC essential to account for redox potentials.

To isolate and study the effect of model size on MSCs we sought to generate models that share the same backbone in terms of subsystems that carry significant fluxes and are central to carbon energy and redox metabolism but differ in the degree of connectivity of these subsystems. The motivation behind this selection for this study lays in our general approach for the systematic generation of context specific models: at first we include all the subsystems that are relevant to the study at hand, and then we investigate to what extent additional levels of connectivity impact the model sensitivity characteristics. The addition of reactions around d-fructose 6-phosphate when expanding from D1 to D2 appeared to significantly affect growth control patterns (Fig. 3A). However, the expansion from D2 to D3 had considerably less impact as top cellular growth FCCs are consistent (Fig. 3B), which suggests that D2 could be a reasonable model choice in terms of complexity for predicting growth control patterns. As d-fructose 6-phosphate is an essential precursor for cell wall fabrication, a network expansion affecting flux distribution around it can be expected to have significant impact on cellular growth control structure. Hence, it is essential to consider the importance of certain metabolic nodes with respect to the study goals in order to ensure no information is lost in the reduction. Again, importance of a metabolic node is strongly influenced by the nature and the objectives of the analysis.

The MCA summation theorem was used to postulate a deviation index (DI) that gave a numerical indication on the consistency of the FCCs with respect to a reaction. Most of the reactions around central carbon metabolism, carrying a higher carbon flux, appeared to have lower DIs. Flux control for reactions with larger fluxes were more robust, particularly if the number of connecting reactions did not change between models for the metabolites participating in the reaction. The larger DIs were noted in the ETC and peripheral reactions. Nevertheless, the consistency in the control patterns of network regions that carry larger carbon flux was consistent across the reduced models when the DI was low, suggesting that their MSC-based predictions are independent of the network complexity. Thus, the DI can be used to study the structural robustness of a kinetic model.

We could argue that the larger the kinetic model is, the more confident and robust the metabolic engineering decisions derived from the model are. However, using our methodology, a modeler can use the DI as an indicator of structural robustness of the system to assess the confidence and quality of the model’s metabolic engineering predictions. In this context we suggest that the experimental design should first target the steps that are consistently better targets across different sized models. Next, we should focus on identifying to what reactions the model predictions are more sensitive to in the larger models, allowing the identification of changes that are responsible for the redistribution of control across the system. Such investigation will serve as a focused analysis for modeling and experiments – understanding which new reactions and pathways, when added during size increase, impact the control distribution in the reference pathways can provide a great insight on how structural changes in a biological network change its function, beyond single enzyme over/down-regulation.

## Conclusions

Failing to preserve certain level of model complexity when constructing kinetic models can result in erroneous conclusions. Model reduction – whether ad hoc or systematic – is a necessary step when constructing kinetic models and could result in false predictions if not done appropriately. To our knowledge, we propose for the first time a workflow for systematically constructing large-scale kinetic models and tailor their size to match the requirements of the studied biological system. We suggested the deviation index as a metric that highlights differences across model MSCs and serves as an indicator in testing kinetic model size adequacy. The nested nature of the reduced models enabled the maintenance of maximum equivalence between the steady-states and kinetic parameters of the populations of kinetic models. This allowed us to assess the impact of model size on the MSC-based conclusions. We showed that despite consistent model reduction and preserving model equivalency, control coefficients can be significantly affected by network size. However, our method can be used to study and assess the adequacy of models based on control coefficients. As systematic model reduction algorithms gain momentum in the field, we hope to pave a path towards building more robust and transferable kinetic models for the community. Classical statistical model assessment tools could be additional avenues to explore when studying structural robustness of models.

## Methods

**Fig. 8:**
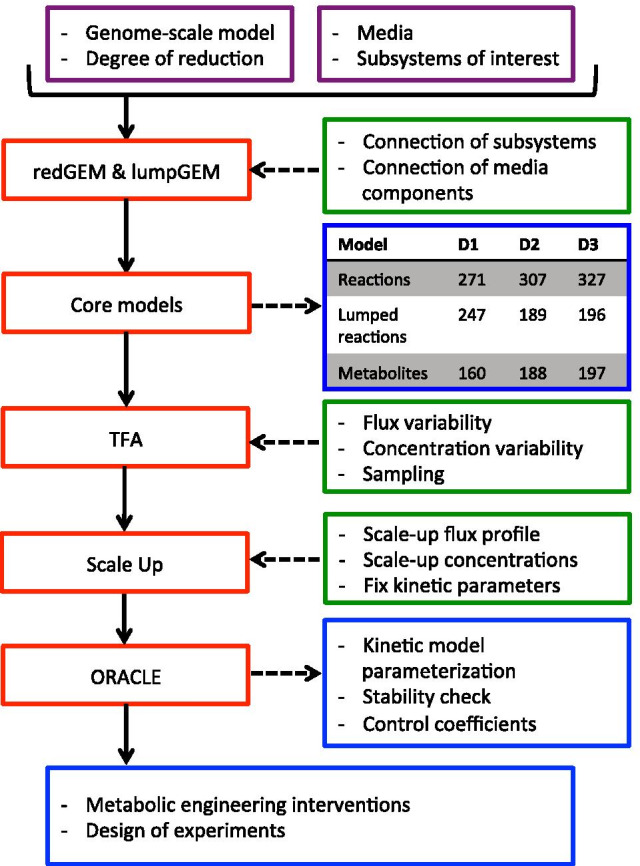
Diagram illustrating the steps carried out in this study. The key steps (red) of the workflow for scaling up and constructing populations of kinetic models with the required inputs (purple). Necessary tasks (green) are completed for each step (red). In the process, outputs (blue) are generated as we move from one key step to the next. Further details about these steps can be found in the below Methods subsections

We developed a workflow for building consistently reduced kinetic models from a genome-scale metabolic model (Fig. 8). We used the redGEM algorithm to construct core models of increasing network size from the *E. coli* iJO1366 genome-scale model. The lumpGEM algorithm was used to generate lumped reactions for the biosynthesis of biomass building blocks (BBBs) for these models. We used thermodynamic-based variability analysis (TVA) [27] to study the flexibility of the models. We proposed a procedure for scaling up the flux and concentration steady-states from one model to another one using the MILP formulation. The ORACLE framework was enhanced, allowing us to keep parametric equivalency between the populations of kinetic models around the steady-states of the reduced models. These steps are further detailed below.

### Model reduction

The stoichiometry of the core networks was defined with the redGEM algorithm, which reduces systematically genome-scale model reconstructions of metabolism [7]. The *E. coli* iJO1366 genome-scale model was reduced, with aerobic minimal media, glucose as the sole carbon source, and the selected starting subsystems corresponding to central carbon metabolism (glycolysis/gluconeogenesis, citric acid cycle, pentose phosphate pathway, pyruvate metabolism, and glyoxylate metabolism). We incorporated all the reactions that use metabolites of the quinone/quinol pools (ubiquinone, ubiquinol, menaquinone, menaquinol, 2-dimethyl menaquinone and 2-dimethyl menaquinol) as the electron transport chain subsystem in order to account for the energy metabolism of the system. redGEM allows the user to define a degree of connection, D, to define the level of connectivity of the core. D is an input parameter of the redGEM and lumpGEM. D corresponds to the number of reaction required to connect the pairs of metabolites between starting subsystems, as defined in [7]. For the purpose of this study we sought to generate nested models that all share the same topology of the central carbon metabolism, but differ only in terms of the reactions that connect the abovementioned subsystems. We generated core networks with a D of 1, 2 and 3, which gave rise to models D1, D2 and D3 respectively. The lumpGEM algorithm [12] was used to generate lumped reactions for the biosynthesis of the BBBs for these core networks. Lumped reactions are sub-networks of reactions composed of non-core reactions that can be used to produce a BBB. Alternative lumped reactions were kept for each of the BBBs. Reactions that could not carry flux were considered as blocked and were removed.

For some of intracellular metabolites, a corresponding transport reaction has not been biochemically characterized and does not appear in the *E. coli* iJO1366 and in our reduced model. However, these metabolites, unless they are highly polar or very large, are subject to passive diffusive transport through the cell membrane. Therefore, we explicitly added transport reactions for these metabolites that operate at least at basal level (10^−6^ mmol/(gDW*h)).

redGEM and lumpGEM algorithms [7, 12] have been made available under the following GitHub repository: https://github.com/EPFL-LCSB/redgem.

### Flux directionality assumptions

As we model the same aerobically grown *E. coli* physiology as in our previous study [35], we make these same directionality assumptions for several bi-directional reactions:

- FBA in mid-lower glycolysis operates towards catabolism [23].
- Magnesium and phosphate transporters are both set towards uptake [24, 25].
- Acetate kinase (ACKr) and phospho-transacetylase (PTAr) operate towards acetate production, as acetate is a by-products [17].
- Succinyl-CoA synthetase (SUCOAS) operates towards producing succinate [17].
- Polyphosphate kinases (PPK2r, and PPKr) are set towards the polyphosphate polymerization [26].

These directionality assumptions were made in agreement with the modeled physiology. Nevertheless, our workflow could be applied to other physiologies and under other directionality assumptions.

### Thermodynamic analysis

The available fluxomics and metabolomics data for the optimal growth of *E. coli* under aerobic conditions and minimal media was integrated in our models. The MILP formulation of the thermodynamics-based flux analysis was used to implement these data into D1, D2 and D3. Since the models were used to build kinetic models, it was undesirable for reactions to be at thermodynamic equilibrium, which would result in them having equal backward and forward fluxes. We imposed MILP constraints to ensure that the thermodynamic displacement, Γ [28, 31, 36], is not at equilibrium. For reactions near equilibrium Γ* ≈* 1. This constraint ensures that we do not have reactions that have net fluxes equal to zero in our system.

Software used for performing thermodynamic-based flux analysis and TVA has been published [22] and is available in MATLAB, and Python 3, on GitHub: respectively, https://github.com/EPFL-LCSB/matTFA and https://github.com/EPFL-LCSB/pytfa.

### Maximum equivalency between steady-states

We sampled the flux space of D1 in order to characterize the solution space without violating physiological, thermodynamic and directionality constraints. The convexity of the solution space enabled us to efficiently sample using the Artificial-Centering Hit-and-Run sampler in the COBRA Toolbox [37, 38]. We sampled 10,000 flux vectors and used Principal Component Analysis (PCA) [39] to select a mean reference state. Nine components covering most of the variance were retained from PCA to select a sample closest to the projected mean. Similarly, we applied this approach for the concentration solution space of this selected flux profile. We selected a unique steady-state for the metabolite concentrations and metabolic fluxes for the demonstration purpose of this study. However, other steady-states could have been used and, in metabolic engineering, alternative steady-states should be considered as they can significantly affect conclusions [35].

In order to make the comparison of the models equitable, we wanted to maintain most similar steady-states between the models. For instance, for D2 we would like the flux vector to be the equal possible to the one from D1. Topological differences in the models make it impossible to have numerically exactly the same flux distribution in larger model for the same reactions. Hence, we take the representative flux from D1 and apply it with percentage relaxation with upper and lower bounds, F^ub^_rxn,i_ and F^lb^_rxn,i_ respectively, into D2. Consequently, we use an MILP formulation to minimize the number of violations of flux boundaries that we are trying to impose. For each intracellular reaction that is shared between the two models we create a binary variable z_rxn, i_ so that when it is equal to 1, the constraints that we impose become inactive. We add for each of these reactions the following constraints:

$$\begin{aligned} NF_{rxn,i} + \left( {F^{ub}_{rxn,i} - UB_{rxn,i} } \right)* \, z_{rxn, \, i} & < \, F^{ub}_{rxn,i} \\ NF_{rxn,i} + \left( {F^{lb}_{rxn,i} - LB_{rxn,i} } \right)* \, z_{rxn, \, i} & > \, F^{lb}_{rxn,i} \\ \end{aligned}$$

where *UB* and *LB* are the upper and lower bounds of the net fluxes *NF* of the reactions. We minimize the sum of the binaries, *z*_*rxn, i*_, in order to have minimal violation of the flux constraints:

Minimize:

$$\mathop \sum \limits_{i}^{\# Fluxes} z_{rxn, i}$$

Subject to:

$$S.v = 0$$

We implied a 1% relaxation to apply and test how many flux constraints we can impose without violation (minimal number of active binary variables *z*_*rxn, i*_). After applying the constraints that are not violating model boundaries of D2, we proceed to sampling the solution space. We selected a sample based on mean PCA as with the representative flux of D1. We then implied in a similar manner the concentration profile from D1 into D2 with a 1% relaxation and sampled the concentration space for this flux profile. We repeat this procedure when scaling up the flux and concentration steady-states from D2 into D3.

### Constructing kinetic models

Populations of kinetic models of metabolism can be constructed with any framework that allows the construction of ensembles of models, as discussed in a recent review [40]. We used the ORACLE framework [2, 3, 28–30, 36, 41–45] to build 50,000 kinetic models around the steady-states for D1, D2 and D3. Available kinetic properties of enzymes from the literature [46] and the databases [47, 48] were incorporated. Reversible Hill kinetics [49] and convenience kinetics [50] were used for reactions with unknown kinetic mechanism (see Additional file 4 for information about kinetic mechanisms and Additional file 7 for their usage across model reactions). Kinetic mechanisms with no or partial information about their parameter values were sampled within the space of kinetic parameters in the form of degree of saturation of enzyme [28]. We parameterized a population of kinetic models, performed consistency tests [28, 42, 51] and computed the MSCs [28, 52]. For further details on the ORACLE workflow the reader is referred to [2, 3, 28–30, 36, 41–45].

We preserved equivalency between populations of kinetic models for D1–3 by fixing the degree of saturation of enzymes from less complex models into the more complex models. We wanted to preserve model equality so that we can fairly compare MSCs of the models. Within the ORACLE framework, we added a feature for fixing the degree of saturation of enzymes. For the parameters that were common between D1 and D2, we fixed the degrees of enzyme saturations from D1 models into D2 models and we sampled the rest of the D2-specific parameters uniformly, until we found a stable model. Hence, we preserved equivalency of the kinetic parameters between D1 and D2. Analogously, we repeated this procedure to imply the degrees of enzymes saturations from D2 into D3. Our stratified sampling approach allows the systematic scaling up and sampling of the parameters that we introduce with each network expansion. Consequently, this stratified sampling approach ensures that we focus on uncertainty introduced by the network expansion alone rather than the other common network parts. Ensuring numerical similarity between the parameters that are shared between two models permits this. Other methods such as a top-down approach of transferring parameters—from D3 to D2, and then from D2 to D1—could be used but this would introduce additional uncertainty from the larger topologies into the smaller networks.

### MSC deviation index

The FCC is a measure of response of a flux to a perturbation in level of enzyme. We compute a FCC, $C_{{p_{k} }}^{{v_{i} }}$, as follows:

$$C_{{p_{k} }}^{{v_{i} }} = \frac{{d lnv_{i} }}{{d lnp_{k} }} = \frac{{p_{k} dv_{i} }}{{v_{i} dp_{k} }}$$

where *v* is the flux across a reaction *i* and *p* is the concentration perturbation of an enzyme *k*.

In MCA the FCCs conform with the summation theorem defined in literature [31, 32]. The theorem implies that all the metabolic fluxes are systemic properties of the model and that their control is shared by all the reactions within the system. The summation theorem makes the assumptions that: (1) the parameters for which we compute flux control coefficients are of first order with respect to the flux, and that (2) the sum of a flux’s control coefficients with respect to all the parameters of the system is equal to one. Hence, the summation theory can be defined as follows [53, 54]:

$$\mathop \sum \limits_{k = 1}^{m} C_{{p_{k} }}^{v} = 1$$

where *m* corresponds to the number of enzymes of the system that can control a flux *v*.

We proposed a deviation index (DI) derived from the summation theorem to quantify the discrepancies in control patterns of a flux between two different models. We define DI as:

$$DI = \left| {\mathop \sum \limits_{k = 1}^{m} C_{{p_{k} }}^{v} - 1} \right|$$

The value of DI will be zero due the summation theory of MCA if we sum over all *m* enzymes of our system. However, if we perform this computation of DI for all the enzymes *m*, except the enzymatic reactions added to the system by a model expansion, we can obtain a deviation from zero. This happens if these enzymatic reactions that were added to the system by the expansion exhibit control over the flux *v* being studied. Hence, if the DI value is not zero for a model expansion, this could suggest that some of the added reactions are important in terms of control to the system.

## Supplementary Information

**Additional file 1.** MATLAB models of D1, D2 and D3. Models used for obtaining thermodynamically feasible steady-states for constructing populations of kinetic models. Software for reading models is available on GitHub: https://github.com/EPFL-LCSB/matTFA.

**Additional file 2.** Fluxomics and metabolomics experimental data incorporated in the model for optimally grown *E. coli*. Table with the flux boundaries in mmol/gDW/h and the concentration boundaries in log(mM) that were applied as constraints to the D1-3 TFA models based on the experimental data reported by McCloskey *et al*.

**Additional file 3.** Thermodynamics-based variability analysis of models. Spreadsheet with the list of metabolites and reactions inside the models with variability analysis of flux and metabolite concentrations. The lumped reactions generated with the redGEM and lumpGEM algorithms begin with LMPD. Reactions added to the *E. coli* iJO1366 for passive diffusive transport start with TransFlux. The rest of the reactions come from *E. coli* iJO1366.

**Additional file 4.** Document with additional figures, details about methods and further analysis carried out in this study. Thermodynamic-based variability analysis was performed to study the feasible ranges of metabolic flux across central carbon reactions for D1-3 models. Flux control coefficients for other reactions were analyzed to see how they are affected by model complexity. Mathematical formulations of the kinetic mechanisms describing the reactions in the kinetic models are also provided.

**Additional file 5.** Flux and concentration steady states. Spreadsheet providing metabolic flux and concentration reference steady-states across the models with a comparative study of these steady states between pairs of models.

**Additional file 6.** Analysis of absolute deviations in means of flux control coefficients for the entire systems. The mean flux control coefficient $C_p^v$ is first computed based on the populations of 50’000 models for D1-3 for all enzymatic reactions with respect to all the enzymes. The absolute deviation between these means of control coefficients is compared pairwise for D1 and D2, and D2 and D3. The vertical corresponds to the reactions *v* and the horizontal to the enzymes *p*. A large deviation indicates a discrepancy in the prediction of the model for that given control coefficient $C_p^v$.

**Additional file 7.** Kinetic mechanism allocation. Reaction mechanisms assigned for the reactions of D1, D2 and D3 models. The assigned reaction mechanisms in this file refer to the kinetic mechanisms for which a mathematical description is given in Additional file 4.

## Data Availability

The datasets generated and/or analysed during the current study are available in the Zenodo repository, https://doi.org/10.5281/zenodo.4587693.

## Abbreviations

ACS: Acetyl-CoA synthase
ADP: Adenosine diphosphate
AKGDH: 2-Oxogluterate dehydrogenase
AMP: Adenosine monophosphate
AMPN: Adenosine monophosphate nucleosidase
ASPTA: Aspartate transaminase
ATP: Adenosine triphosphate
ATPM: Adenosine triphosphate maintenance
BBB: Biomass building block
COBRA: Constraint-based reconstruction and analysis
DI: Deviation index
DRUM: Dynamic reduction of unbalanced metabolism
ETC: Electron transport chain
FBA: Flux balance analysis
FCC: Flux control coefficient
GEM: Genome-scale metabolic reconstruction
GNK: Gluconokinase
MCA: Metabolic control analysis
MILP: Mixed integer linear programming
MSC: Metabolic sensitivity coefficients
NAD: Nicotinamide adenine dinucleotide
NADPH: Nicotinamide adenine dinucleotide phosphate hydrogen
ORACLE: Optimization and risk analysis of complex living entities
PCA: Principal component analysis
PFK: Phosphofructokinase
PGI: Glucose-6-phosphate isomerase
PGK: Phosphoglycerate kinase
PGM: Phosphoglycerate mutase
PPCK: Phosphoenolpyruvate carboxykinase
PPP: Pentose phosphate pathway
PPS: Phosphoenolpyruvate synthase
PSCVT: Polyenolpyruvate to 3-phosphoshikimate 1-carboxyvinyltransferase
PYK: Pyruvate kinase
RPE: Ribulose 5-phosphate 3-epimerase
RPI: Ribose-5-phosphate isomerase
SUCOAS: Succinyl-CoA synthetase
TALA: Transaldolase
TCA: Tricarboxylic acid
TPI: Triosephosphate isomerase
TVA: Thermodynamic-based variability analysis
