# Supplementary material for: The effects of model complexity and size on metabolic flux distribution and control: case study in Escherichia coli
Source: BMC Bioinformatics. 2021 Mar 20;22:134. doi: 10.1186/s12859-021-04066-y (PMC7981984; doi:10.1186/s12859-021-04066-y)
Supplement: Supplementary file 4 — Additional file 4. Document with additional figures, details about methods and further analysis carried out in this study. Thermodynamic-based variability analysis was performed to study the feasible ranges of metabolic flux across central carbon reactions for D1-3 models. Flux control coefficients for other reactions were analyzed to see how they are affected by model complexity. Mathematical formulations of the kinetic mechanisms describing the reactions in the kinetic models are also provided. [file 12859_2021_4066_MOESM4_ESM.docx]

**Additional File 4 – Supporting Information**

**Hameri et al.**

## Thermodynamic-based variability analysis

Analysis of reaction flux ranges from TVA for D1, D2 and D3 revealed several considerable differences between the central carbon reactions. When comparing the ranges between D1 and D2, the largest differences were noted in adenylate kinase (ADK1), tartronate semialdehyde reductase (TRSARr), glucose-6-phosphate isomerase (PGI), phosphoglucomutase (PGMT), D-lactate dehydrogenase (LDH_D), triose-phosphate isomerase (TPI), phosphoglycerate kinase (PGK), glyceraldehyde-3-phosphate dehydrogenase (GAPD), phosphoglycerate mutase (PGM) and enolase (ENO) (S2 Table). Performing the same analysis between D2 and D3 reveals that only transketolase (TKT2) and glutamate dehydrogenase (GLUDy) changed considerably in ranges amongst central carbon reactions. In fact, GLUDy became bidirectional with this expansion. Other differences were noted in the peripheral reactions pertaining to additions from expansion from D2 to D3. Generally, most differences in the flux variability ranges resulted from the bypass routes that additional reactions provided, which resulted in certain reactions becoming bi-directional (Figure 1).

The TVA was also performed on concentration ranges of the models and we notice several differences in the allowable ranges of metabolite concentrations between D1, D2 and D3. Most noticeable concentration range differences between D1 and D2 occurred in D-glucose 6-phosphate, D-fructose 6-phosphate, fumarate, L-arinine and S-Dihydroorotate (S2 Table and Figure SI below). However, between D2 and D3, noticeable differences were only noted in the ranges of D-glucose 6-phosphate and D-fructose 6-phosphate. The comparison of these TVA ranges in metabolite concentrations and reaction fluxes reveals that more considerable differences occurred between D1 and D2 than between D2 and D3.

**Supplemental figures**

**
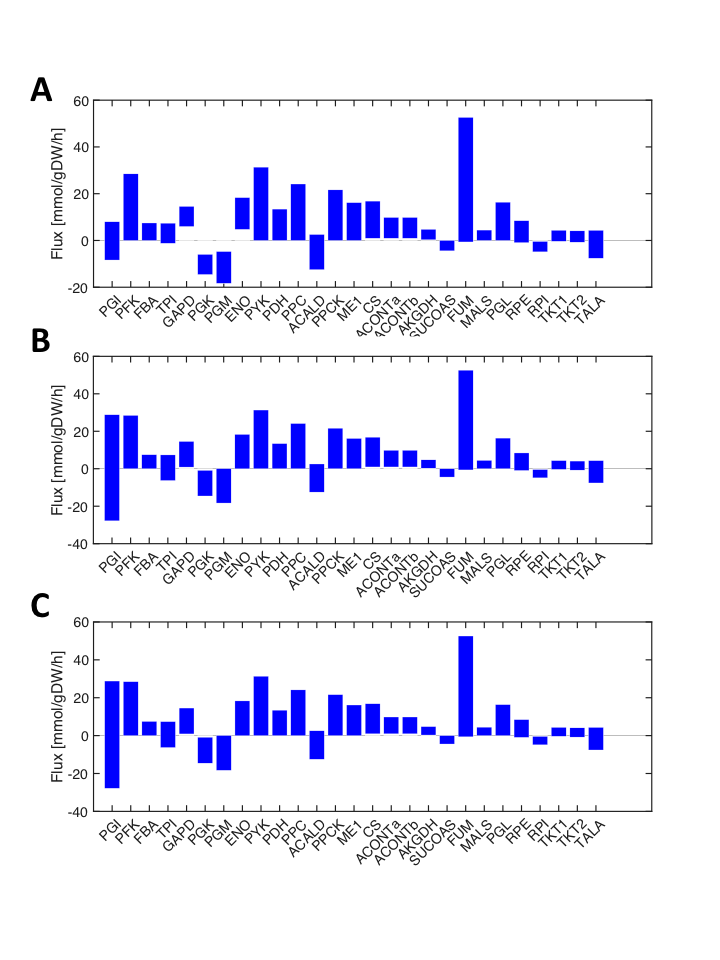
**

**Figure SI.** **Flux variability analysis around central carbon reactions.** Bar plots highlighting thermodynamically feasible flux ranges of D1 **(a)**, D2 **(b)** and D3 **(c)**.

**Figure SII.** **Flux control coefficients of growth with respect ATPM.** Scatter plot comparing the control of growth with respect to ATPM for 48’080 stable pairs of kinetic models for D1 and D2.

**
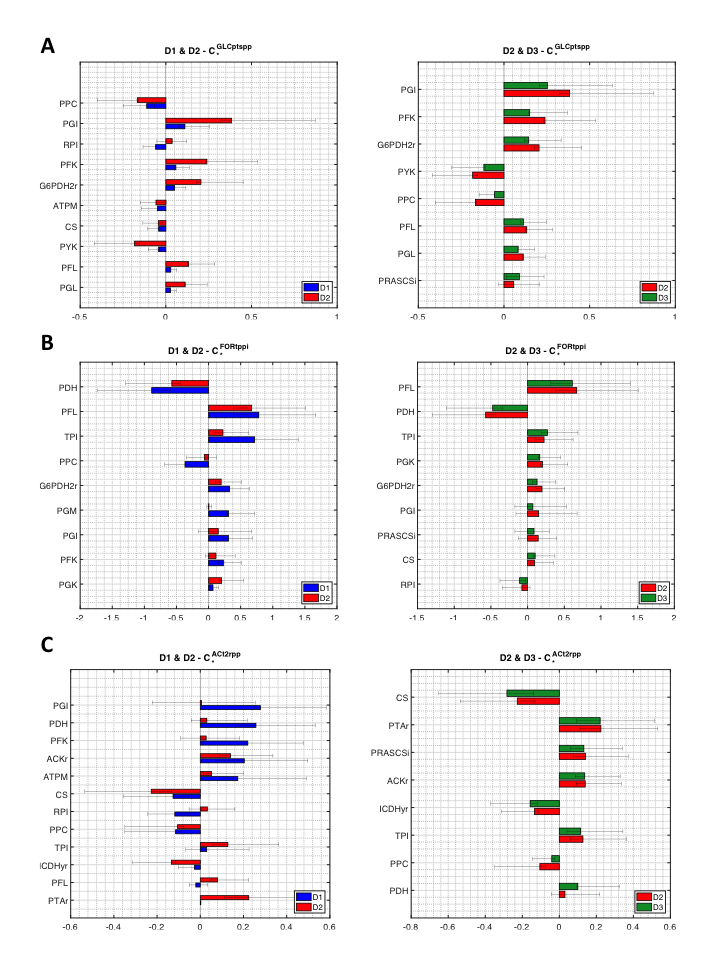
**

**Figure SIII.** **Flux control coefficients of (a) glucose uptake, (b) formate excretion and (c) acetate excretion across the models.** Pairwise illustration of the union of the top 7 enzymes across the models in terms of absolute control over cellular growth for D1 versus D2, and D2 versus D3. The whiskers give the upper and lower quartiles of the FCC populations and the bars give the means.

**Kinetic mechanisms**

These are the kinetic mechanisms that were used to construct the populations of kinetic models. The allocation of these mechanisms to reactions in the D1, D2 and D3 models is provided in S5 Table.

**A1**

Uni-uni reversible Michaelis-Menten kinetic mechanism:

$$S\begin{matrix} k_{f} \\ \rightleftarrows\\ k_{b} \end{matrix}P$$

$$V_{net}=V_{f}-V_{b}=V_{{max}_{f}}\frac{\tilde{S}}{\tilde{S}+\tilde{P}+1}{-V}_{{max}_{b}}\frac{\tilde{P}}{\tilde{S}+\tilde{P}+1}$$

where $\tilde{S}$ and $\tilde{P}$ are the scaled concentrations of the substrate and the product, respectively as follows:

$$\tilde{S}=\frac{[S]}{K_{M_{S}}}$$

$$\tilde{P}=\frac{[P]}{K_{M_{P}}}$$

The elasticities are computed as follows:

$$\varepsilon_{f,s}=\frac{1+\tilde{P}}{1+\tilde{S}+\tilde{P}}$$

$$\varepsilon_{f,p}=\frac{-\tilde{P}}{1+\tilde{S}+\tilde{P}}$$

$$\varepsilon_{b,s}=\frac{-\tilde{S}}{1+\tilde{S}+\tilde{P}}$$

$$\varepsilon_{b,p}=\frac{1+\tilde{S}}{1+\tilde{S}+\tilde{P}}$$

**A1CSP**

This is an A1 mechanism where the elasticities of the substrate and product are calculated as was done in A1. However, we have a compensating substrate C_S_ and compensating product C_P_ participating:

$$S+C_{S}\begin{matrix} k_{f} \\ \rightleftarrows\\ k_{b} \end{matrix}P+C_{P}$$

We modeled the elasticities for these compensating metabolites as:

$$\varepsilon_{f,Cs}=1$$

$$\varepsilon_{f,Cp}=0$$

$$\varepsilon_{b,Cs}=0$$

$$\varepsilon_{b,Cp}=1$$

**A1CS2P2**

This is an A1 mechanism where we have two compensating substrates (C_S1_ and C_S2_) and two compensating product (C_P1_ and C_P2_) participating:

$$S+C_{S1}+C_{S2}\begin{matrix} k_{f} \\ \rightleftarrows\\ k_{b} \end{matrix}P+C_{P1}+C_{P2}$$

We modeled the elasticities for these compensating metabolites as:

$$\varepsilon_{f,Cs1}=1$$

$$\varepsilon_{f,Cs2}=1$$

$$\varepsilon_{f,Cp1}=0$$

$$\varepsilon_{f,Cp2}=0$$

$$\varepsilon_{b,Cs1}=0$$

$$\varepsilon_{b,Cs2}=0$$

$$\varepsilon_{b,Cp1}=1$$

$$\varepsilon_{b,Cp2}=1$$

**A1CP2i**

The elasticities for the substrate and product are the same as in A1 but this time we have two compensating products (C_P1_ and C_P2_):

$$S\begin{matrix} k_{f} \\ \rightleftarrows\\ k_{b} \end{matrix}P+C_{P1}+C_{P2}$$

The elasticities are computed as follows for the compensating products:

$$\varepsilon_{f,Cp1}=0$$

$$\varepsilon_{f,Cp2}=0$$

$$\varepsilon_{b,Cp1}=1$$

$$\varepsilon_{b,Cp2}=1$$

**A2**

This is a generalized reversible Hill mechanism with two substrates (S_1_ and S_2_) and two products (P_1_ and P_2_) participating for a hill coefficient of 1:

$$S_{1}+S_{2}\begin{matrix} k_{f} \\ \rightleftarrows\\ k_{b} \end{matrix}P_{1}+P_{2}$$

$$V_{net}=V_{f}-V_{b}=V_{{max}_{f}}\frac{\tilde{S_{1}}\tilde{S_{2}}}{\left( \tilde{S_{1}}+\tilde{P_{1}}+1 \right)\left( \tilde{S_{2}}+\tilde{P_{2}}+1 \right)}{-V}_{{max}_{b}}\frac{\tilde{P_{1}}\tilde{P_{2}}}{\left( \tilde{S_{1}}+\tilde{P_{1}}+1 \right)\left( \tilde{S_{2}}+\tilde{P_{2}}+1 \right)}$$

where the ~ above the substrate and products indicates that they are scaled concentrations:

$$\tilde{S_{1}}=\frac{[S_{1}]}{K_{M_{S1}}}$$

$$\tilde{S_{2}}=\frac{[S_{2}]}{K_{M_{S2}}}$$

$$\tilde{P_{1}}=\frac{[P_{1}]}{K_{M_{P1}}}$$

$$\tilde{P_{2}}=\frac{[P_{2}]}{K_{M_{P2}}}$$

We compute the elasticities with respect to the substrates and the products as:

$$\varepsilon_{f,s1}=\frac{1+\tilde{P_{1}}}{1+\tilde{S_{1}}+\tilde{P_{1}}}$$

$$\varepsilon_{f,s2}=\frac{1+\tilde{P_{2}}}{1+\tilde{S_{2}}+\tilde{P_{2}}}$$

$$\varepsilon_{f,p1}=\frac{-\tilde{P_{1}}}{1+\tilde{S_{1}}+\tilde{P_{1}}}$$

$$\varepsilon_{f,p2}=\frac{-\tilde{P_{2}}}{1+\tilde{S_{2}}+\tilde{P_{2}}}$$

$$\varepsilon_{b,s1}=\frac{-\tilde{S_{1}}}{1+\tilde{S_{1}}+\tilde{P_{1}}}$$

$$\varepsilon_{b,s2}=\frac{-\tilde{S_{2}}}{1+\tilde{S_{2}}+\tilde{P_{2}}}$$

$$\varepsilon_{b,p1}=\frac{1+\tilde{S_{1}}}{1+\tilde{S_{1}}+\tilde{P_{1}}}$$

$$\varepsilon_{b,p2}=\frac{1+\tilde{S_{2}}}{1+\tilde{S_{2}}+\tilde{P_{2}}}$$

**A2CSP**

In this mechanism we model the substrate and product elasticities like in the A2 mechanism. However, we compensate for one substrate $C_{S}$and one product $C_{P}$.

$$S_{1}+S_{2}+C_{S}\begin{matrix} k_{f} \\ \rightleftarrows\\ k_{b} \end{matrix}P_{1}+P_{2}+C_{P}$$

We modeled the elasticities for these compensating metabolites as:

$$\varepsilon_{f,Cs}=1$$

$$\varepsilon_{f,Cp}=0$$

$$\varepsilon_{b,Cs}=0$$

$$\varepsilon_{b,Cp}=1$$

**A2CS2P2**

In this mechanism we model the substrate and product elasticities like in the A2 mechanism. However, we compensate for two substrates (C_S1_ and C_S2_) and two products (C_P1_ and C_P2_).

$$S_{1}+S_{2}+C_{S1}+C_{S2}\begin{matrix} k_{f} \\ \rightleftarrows\\ k_{b} \end{matrix}P_{1}+P_{2}+C_{P1}+C_{P2}$$

We modeled the elasticities for these compensating metabolites as:

$$\varepsilon_{f,Cs1}=1$$

$$\varepsilon_{f,Cs2}=1$$

$$\varepsilon_{f,Cp1}=0$$

$$\varepsilon_{f,Cp2}=0$$

$$\varepsilon_{b,Cs1}=0$$

$$\varepsilon_{b,Cs2}=0$$

$$\varepsilon_{b,Cp1}=1$$

$$\varepsilon_{b,Cp2}=1$$

**RHUB**

This is a reversible Hill kinetic approximation of the uni-bi kinetic mechanism:

$$S\begin{matrix} k_{f} \\ \rightleftarrows\\ k_{b} \end{matrix}P_{1}+P_{2}$$

$$V_{net}=V_{f}-V_{b}=V_{{max}_{f}}\frac{\tilde{S}}{\left( 1+\tilde{S}+\tilde{P_{1}}+\tilde{P_{2}}+\tilde{P_{1}}\tilde{P_{2}} \right)}{-V}_{{max}_{b}}\frac{\tilde{P_{1}}\tilde{P_{2}}}{\left( 1+\tilde{S}+\tilde{P_{1}}+\tilde{P_{2}}+\tilde{P_{1}}\tilde{P_{2}} \right)}$$

We compute the elasticities for the mechanism as follows:

$$\varepsilon_{f,s}=1-\frac{\tilde{S}}{\left( 1+\tilde{S}+\tilde{P_{1}}+\tilde{P_{2}}+\tilde{P_{1}}\tilde{P_{2}} \right)}$$

$$\varepsilon_{f,p1}=\frac{-\tilde{P_{1}}(1+\tilde{P_{2}})}{\left( 1+\tilde{S}+\tilde{P_{1}}+\tilde{P_{2}}+\tilde{P_{1}}\tilde{P_{2}} \right)}$$

$$\varepsilon_{f,p2}=\frac{-\tilde{P_{2}}(1+\tilde{P_{1}})}{\left( 1+\tilde{S}+\tilde{P_{1}}+\tilde{P_{2}}+\tilde{P_{1}}\tilde{P_{2}} \right)}$$

$$\varepsilon_{b,s}=\frac{-\tilde{S}}{\left( 1+\tilde{S}+\tilde{P_{1}}+\tilde{P_{2}}+\tilde{P_{1}}\tilde{P_{2}} \right)}$$

$$\varepsilon_{b,p1}=1-\frac{\tilde{P_{1}}(1+\tilde{P_{2}})}{\left( 1+\tilde{S}+\tilde{P_{1}}+\tilde{P_{2}}+\tilde{P_{1}}\tilde{P_{2}} \right)}$$

$$\varepsilon_{b,p2}=1-\frac{\tilde{P_{2}}(1+\tilde{P_{1}})}{\left( 1+\tilde{S}+\tilde{P_{1}}+\tilde{P_{2}}+\tilde{P_{1}}\tilde{P_{2}} \right)}$$

**RHUBCSP**

The elasticities for the substrate and products are the same as for the RHUB mechanism but we have a compensating substrate $C_{S}$and a compensating product$C_{P}$.

$$S+C_{S}\begin{matrix} k_{f} \\ \rightleftarrows\\ k_{b} \end{matrix}P_{1}+P_{2}+C_{P}$$

We modeled the elasticities for these compensating metabolites as:

$$\varepsilon_{f,Cs}=1$$

$$\varepsilon_{f,Cp}=0$$

$$\varepsilon_{b,Cs}=0$$

$$\varepsilon_{b,Cp}=1$$

**RHUBCS2P2**

The elasticities for the substrate and products are the same as for the RHUB mechanism. However, we compensate for two substrates (C_S1_ and C_S2_) and two products (C_P1_ and C_P2_).

$$S+C_{S1}+C_{S2}\begin{matrix} k_{f} \\ \rightleftarrows\\ k_{b} \end{matrix}P_{1}+P_{2}+C_{P1}+C_{P2}$$

We modeled the elasticities for these compensating metabolites as:

$$\varepsilon_{f,Cs1}=1$$

$$\varepsilon_{f,Cs2}=1$$

$$\varepsilon_{f,Cp1}=0$$

$$\varepsilon_{f,Cp2}=0$$

$$\varepsilon_{b,Cs1}=0$$

$$\varepsilon_{b,Cs2}=0$$

$$\varepsilon_{b,Cp1}=1$$

$$\varepsilon_{b,Cp2}=1$$

**RHBU**

This is a reversible Hill kinetic approximation of the bi-uni kinetic mechanism:

$$S_{1}+S_{2}\begin{matrix} k_{f} \\ \rightleftarrows\\ k_{b} \end{matrix}P$$

$$V_{net}=V_{f}-V_{b}=V_{{max}_{f}}\frac{\tilde{S_{1}}\tilde{S_{2}}}{\left( 1+\tilde{P}+\tilde{S_{1}}+\tilde{S_{2}}+\tilde{S_{1}}\tilde{S_{2}} \right)}{-V}_{{max}_{b}}\frac{\tilde{P}}{\left( 1+\tilde{P}+\tilde{S_{1}}+\tilde{S_{2}}+\tilde{S_{1}}\tilde{S_{2}} \right)}$$

We compute the elasticities for the substrates and product as follows:

$$\varepsilon_{f,s1}=1-\frac{\tilde{S_{1}}(1+\tilde{S_{2}})}{\left( 1+\tilde{P}+\tilde{S_{1}}+\tilde{S_{2}}+\tilde{S_{1}}\tilde{S_{2}} \right)}$$

$$\varepsilon_{f,s2}=1-\frac{\tilde{S_{2}}(1+\tilde{S_{1}})}{\left( 1+\tilde{P}+\tilde{S_{1}}+\tilde{S_{2}}+\tilde{S_{1}}\tilde{S_{2}} \right)}$$

$$\varepsilon_{f,p}=\frac{-\tilde{P}}{\left( 1+\tilde{P}+\tilde{S_{1}}+\tilde{S_{2}}+\tilde{S_{1}}\tilde{S_{2}} \right)}$$

$$\varepsilon_{b,s1}=-\frac{\tilde{S_{1}}(1+\tilde{S_{2}})}{\left( 1+\tilde{P}+\tilde{S_{1}}+\tilde{S_{2}}+\tilde{S_{1}}\tilde{S_{2}} \right)}$$

$$\varepsilon_{b,s2}=-\frac{\tilde{S_{2}}(1+\tilde{S_{1}})}{\left( 1+\tilde{P}+\tilde{S_{1}}+\tilde{S_{2}}+\tilde{S_{1}}\tilde{S_{2}} \right)}$$

$$\varepsilon_{b,p}=1-\frac{-\tilde{P}}{\left( 1+\tilde{P}+\tilde{S_{1}}+\tilde{S_{2}}+\tilde{S_{1}}\tilde{S_{2}} \right)}$$

**RHBUCSP**

This is a reversible Hill kinetic approximation of the bi-uni kinetic mechanism (RHBU) with compensation for a substrate $C_{S}$ and a product $C_{P}$:

$$S_{1}+S_{2}+C_{S}\begin{matrix} k_{f} \\ \rightleftarrows\\ k_{b} \end{matrix}P+C_{P}$$

We modeled the elasticities for these compensating metabolites as:

$$\varepsilon_{f,Cs}=1$$

$$\varepsilon_{f,Cp}=0$$

$$\varepsilon_{b,Cs}=0$$

$$\varepsilon_{b,Cp}=1$$

**RHBUCS2P2**

This is a reversible Hill kinetic approximation of the bi-uni kinetic mechanism (RHBU) where we compensate for two substrates (C_S1_ and C_S2_) and two products (C_P1_ and C_P2_):

$$S_{1}+S_{2}+C_{S1}+C_{S2}\begin{matrix} k_{f} \\ \rightleftarrows\\ k_{b} \end{matrix}P+C_{P1}+C_{P2}$$

We modeled the elasticities for these compensating metabolites as:

$$\varepsilon_{f,Cs1}=1$$

$$\varepsilon_{f,Cs2}=1$$

$$\varepsilon_{f,Cp1}=0$$

$$\varepsilon_{f,Cp2}=0$$

$$\varepsilon_{b,Cs1}=0$$

$$\varepsilon_{b,Cs2}=0$$

$$\varepsilon_{b,Cp1}=1$$

$$\varepsilon_{b,Cp2}=1$$

**A99**

Bi-bi reversible hill reaction mechanism where PEP is the external inhibitor (M) of the PFK reaction:

$$S_{1}+S_{2}\begin{matrix} k_{f} \\ \rightleftarrows\\ k_{b} \end{matrix}P_{1}+P_{2}$$

$$V_{net}=V_{f}-V_{b}$$

$$=V_{{max}_{f}}\frac{\tilde{S_{1}}\tilde{S_{2}}\left( \tilde{S_{1}}+\tilde{P_{1}} \right)^{3}\left( \tilde{S_{2}}+\tilde{P_{2}} \right)^{3}}{D}{-V}_{{max}_{b}}\frac{\tilde{P_{1}}\tilde{P_{2}}\left( \tilde{S_{1}}+\tilde{P_{1}} \right)^{3}\left( \tilde{S_{2}}+\tilde{P_{2}} \right)^{3}}{D}$$

$$D=\left( \tilde{S_{1}}+\tilde{P_{1}} \right)^{4}\left( \tilde{S_{2}}+\tilde{P_{2}} \right)^{4}$$

$$+\frac{\left( \tilde{M}^{4}+1 \right)\left( {\tilde{P_{2}}}^{4}+1 \right)+\left( \left( \tilde{S_{1}}+\tilde{P_{1}} \right)^{4}+\left( \tilde{S_{2}}+\tilde{P_{2}} \right)^{4} \right)\left( \alpha\tilde{M}^{4}+1 \right)\left( \beta{\tilde{P_{2}}}^{4}+1 \right)}{\left( \alpha^{2}\tilde{M}^{4}+1 \right)\left( \beta^{2}{\tilde{P_{2}}}^{4}+1 \right)}$$

where the modifier scaled concentration is:

$$\tilde{M}=\frac{[M]}{K_{M_{M}}}$$

and the effects of the inhibition by PEP and the activation by ADP are modeled by

$$\alpha=0.01$$

$$\beta=100$$

The elasticities can be derived as demonstrated previously in work done by Andreozzi et al [1].

**CONVKIN2**

The convenience kinetic rate approximation for general stoichiometries such as:

$${\alpha_{1}S}_{1}+{\alpha_{2}S}_{2}+\ldots\begin{matrix} k_{f} \\ \rightleftarrows\\ k_{b} \end{matrix}{\beta_{1}P}_{1}+{\beta_{2}P}_{2}+\ldots$$

is given by the following approximation:

$$V_{net}=V_{f}-V_{b}{=V}_{{max}_{f}}\frac{\prod_{i} {\tilde{S_{i}}}^{\alpha_{i}}}{\prod_{i} \left( 1+\tilde{S_{i}}+\ldots+{\tilde{S_{i}}}^{\alpha_{i}} \right)+\prod_{j} \left( 1+\tilde{P_{j}}+\ldots+{\tilde{P_{j}}}^{\beta_{j}} \right)-1}$$

$${-V}_{{max}_{b}}\frac{\prod_{j} {\tilde{P_{j}}}^{\beta_{j}}}{\prod_{i} \left( 1+\tilde{S_{i}}+\ldots+{\tilde{S_{i}}}^{\alpha_{i}} \right)+\prod_{j} \left( 1+\tilde{P_{j}}+\ldots+{\tilde{P_{j}}}^{\beta_{j}} \right)-1}$$

The elasticities can be obtained for general stoichiometry and for any possible coefficients of substrates and products. The ~ above the substrate S and products P indicates that they are scaled concentrations.

**GENCHEM**

The mass action kinetics for general stoichiometries such as:

$$c_{S1}S_{1}+c_{S2}S_{2}+\ldots+c_{Sn}S_{nS}\begin{matrix} k_{f} \\ \rightleftarrows\\ k_{r} \end{matrix}c_{P1}P_{1}+c_{P2}P_{2}+\ldots+c_{Pn}P_{nP}$$

is given by the following approximation:

$$V_{net}=V_{f}-V_{r}=k_{f}\left[ S_{1} \right]^{c_{S1}}\left[ S_{2} \right]^{c_{S2}}\ldots\left[ S_{n} \right]^{c_{SnS}}-k_{r}\left[ P_{1} \right]^{c_{P1}}\left[ P_{2} \right]^{c_{P2}}\ldots\left[ P_{n} \right]^{c_{PnP}}$$

The elasticities can be obtained for general stoichiometry and for any possible coefficients of substrates and products.

**IRRX**

Irreversible reactions are modeled with irreversible Michaelis-Menten kinetics. For example the reaction below:

$${3S}_{1}+S_{2}\begin{matrix} k_{f} \\ \to\\ \end{matrix}{4P}_{1}+P_{2}$$

would be modeled as:

$$V_{net}=V_{f}-V_{b}=V_{{max}_{f}}\frac{\tilde{S_{1}}\tilde{S_{2}}}{\left( \tilde{S_{1}}+1 \right)\left( \tilde{S_{2}}+1 \right)}-0=V_{{max}_{f}}\frac{\tilde{S_{1}}\tilde{S_{2}}}{\left( \tilde{S_{1}}+1 \right)\left( \tilde{S_{2}}+1 \right)}$$

The elasticities can be obtained for general stoichiometry and for any possible coefficients of substrates and products. The stoichiometric coefficients do not take part into the kinetic rate expression for this mechanism.

**References**

1. Andreozzi S, Chakrabarti A, Soh KC, Burgard A, Yang TH, Van Dien S, et al. Identification of metabolic engineering targets for the enhancement of 1,4-butanediol production in recombinant E. coli using large-scale kinetic models. Metabolic engineering. 2016;35:148-59. doi: 10.1016/j.ymben.2016.01.009. PubMed PMID: 26855240.
